# Local erythropoietin and endothelial progenitor cells improve regional cardiac function in acute myocardial infarction

**DOI:** 10.1186/1471-2261-10-43

**Published:** 2010-09-17

**Authors:** Andreas Stein, Martina Knödler, Markus Makowski, Sandra Kühnel, Stefan Nekolla, Alexandra Keithahn, Eliane Weidl, Philip Groha, Maren Schürmann, Atti Saraste, Rene Botnar, Robert AJ Oostendorp, Ilka Ott

**Affiliations:** 1 Deutsches Herzzentrum der Technischen Universität München, Lazarettstr. 36, 80636 München, Germany; 2 Medizinische Klinik der Technischen Universität München, Ismaningerstr. 22, 81675 München, Germany; 3 Nuklearmedizinische Klinik und Poliklinik der Technischen Universität München, Ismaningerstr. 22, 81675 München, Germany

## Abstract

**Background:**

Expanded endothelial progenitor cells (eEPC) improve global left ventricular function in experimental myocardial infarction (MI). Erythropoietin beta (EPO) applied together with eEPC may improve regional myocardial function even further by anti-apoptotic and cardioprotective effects. Aim of this study was to evaluate intramyocardial application of eEPCs and EPO as compared to eEPCs or EPO alone in experimental MI.

**Methods and Results:**

In vitro experiments revealed that EPO dosed-dependently decreased eEPC and leukocyte apoptosis. Moreover, in the presence of EPO mRNA expression in eEPC of proangiogenic and proinflammatory mediators measured by TaqMan PCR was enhanced. Experimental MI was induced by ligation and reperfusion of the left anterior descending coronary artery of nude rats (n = 8-9). After myocardial transplantation of eEPC and EPO CD68+ leukocyte count and vessel density were enhanced in the border zone of the infarct area. Moreover, apoptosis of transplanted CD31 + TUNEL + eEPC was decreased as compared to transplantation of eEPCs alone. Regional wall motion of the left ventricle was measured using Magnetic Resonance Imaging. After injection of eEPC in the presence of EPO regional wall motion significantly improved as compared to injection of eEPCs or EPO alone.

**Conclusion:**

Intramyocardial transplantation of eEPC in the presence of EPO during experimental MI improves regional wall motion. This was associated with an increased local inflammation, vasculogenesis and survival of the transplanted cells. Local application of EPO in addition to cell therapy may prove beneficial in myocardial remodeling.

## Background

Bone marrow derived progenitor cells are mobilized to the blood in acute myocardial infarction[1-3]. After recruitment to the ischemic myocardium they contribute to regeneration by neovascularization and release of paracrine mediators[4-6]. Similarly application of progenitor cells after myocardial infarction has been shown to improve cardiac function in experimental and clinical studies [7,8]. Analysis of survival of the applied cells demonstrated that the vast majority of cells vanished shortly after transplantation[9]. Therefore additional treatments to improve the survival of cells may increase their regenerative capacity and improve myocardial function after myocardial infarction.

Besides its haematopoietic effects erythropoietin (EPO) has anti-apoptotic effects especially under ischemic conditions and attenuates oxidative stress[10-14]. Erythropoietin receptors are not only expressed on erythroid precursors, but also on megakaryocytes, vascular smooth muscle cells, endothelial cells, skeletal myoblasts, neurons, nephrons, and cardiac myocytes. EPO binding to the EPO receptor leads to a homodimerization, with subsequent activation of janus kinase 2[15-18]. EPO signaling involves multiple pathways including activation of STAT 5, activation of proteins with Src homology 2 domains, such as PI3 kinase and activation of ras/MAP kinases[19]. Proangiogenic properties of EPO and subsequent cell proliferation and differentiation were observed in vitro after stimulation of cultured endothelial cells with EPO. Furthermore improved wound healing and angiogenesis was found in mice after application of EPO [20-22]. Moreover systemic application of EPO mobilizes progenitor cells from the bone marrow to the peripheral blood, which was associated with an improved myocardial function after myocardial infarction in mice[23,24].

Recent studies have shown that intramyocardial injection of expanded endothelial progenitor cells (eEPC) improve global ejection fraction in experimental myocardial infarction (MI) [1].

Aim of this study was to investigate if addition of EPO to the transplanted eEPC improves regional wall motion and to investigate the survival of eEPC in the presence and absence of EPO. As potential mechanisms to alter regional wall motion the local inflammatory response and vasculogenesis were analyzed.

## Methods

### Expansion of EPC (eEPC) and effect of EPO on apoptosis of mononuclear leukocytes and eEPC in vitro

Mononuclear cells were isolated from human umbilical cord blood by density gradient centrifugation. CD34+ cells were isolated using immunomagnetic-beads. CD34+ cord blood cells were cultured in endothelial medium and expanded to passage 4 and 5 as described (eEPCs)[6].

Leukocyte suspensions were prepared by dextran sedimentation and hypotonic lysis as described [25]. Apoptosis of eEPCs was analyzed in an in vitro apoptosis assay (Annexin-V-Fluostaining Kit, Roche, Mannheim, Germany) after H_2_O_2 _stimulation for 24 hours and of leukocytes after serum starvation for 8 hours in the absence and presence of EPO with the concentrations as indicated (NeoRecormon^® ^Multidose, Roche, Mannheim). Apoptosis was quantified by flow cytometry after annexin staining.

### Effects of EPO on mRNA expression in vitro

RNA of leukocytes and eEPCs after stimulation with 200 IU/ml EPO for 3 hours and without stimulation was extracted by Trizol Reagent (Trizol # 15596-026, Invitrogen, Germany) according to manufacturer instructions and cDNA was synthesized as described previously. Quantitative TaqMan PCR was performed using Human Inflammatory Cytokines and Receptors PCR Array and Angiogenesis PCR Array (PAHS-011, PAHS-024, SABiosciences, Frederick, USA). Assays-on-Demand containing specific primers and probe for Bax (Hs00180269_m1) and GAPDH (Hs99999905_m1) were from Applied Biosystems. Relative RNA expression was calculated using the ΔΔC_t_-method by normalization on GAPDH.

### Experimental myocardial infarction

Two models of experimental myocardial infarction were utilized. To establish the method of measuring regional wall motion by MRI in large myocardial infarcts we performed a permanent ligation model of the left anterior descending artery (Lig, n = 8). Since ischemia reperfusion is more relevant for the clinical situation the effects of eEPC in the presence and absence of EPO on regional wall motion were analyzed using a model of reperfused myocardial infarction. Myocardial ischemia was induced by temporary ligation of the left anterior descending coronary artery for 30 minutes in male athymic nude rats (CRL:NIH-rnu, Charles River Laboratories, Sulzfeld, Germany). After reperfusion was initiated by release of the ligation 1×10^6 ^eEPC cells in 150 μl PBS (eEPC n = 9), 1×10^6 ^eEPC cells in 150 μl PBS containaing 200 U EPO (eEPC + Epo, n = 9), 150 μl PBS containg 200 U EPO (Epo, n = 8) or PBS alone (control, n = 8) were injected intramyocardial with a 27-gauge needle as described [1]. To avoid volume disturbances at the local site of injection and to allow a wider distribution of the transplanted cells, 30 μl injections comprising 2 × 10^5 ^cells per injection site were performed into 5 sites at distances of about 60° into the borderzone of the ischemic area. The ischemic zone was identified by the pale color of the myocardium. Anesthesia was induced using medetomidin (150 μg/kg), midazolam (2 mg/kg), and fentanyl (5 μg/kg) and was antagonized using atipamezol (0.75 mg/kg), flumazenil (200 μg/kg), and naloxon (120 μg/kg). After 4 weeks cardiac MRI was performed to assess myocardial function. Subsequently, rats were sacrificed by an overdose of pentobarbital and hearts were fixed in 4% paraformaldehyde, embedded in paraffin and sliced into transverse sections. Three groups were designed as follows: ischemia and reperfusion (IR; n = 8), permanent ligation (Lig; n = 8) and sham operation (Sham, n = 6). An additional 5 rats in each group were sacrificed after 3 days for histological analysis. Áll studies were approved by the institutional Animal Care and Use Committee (Bayerisches Wissenschaftsministerium).

### Magnetic resonance imaging

MRI was performed on a clinical 1.5 Tesla Philips Achieva MR tomograph (Philips Medical System, Best, The Netherlands) with a small 47 mm flex loop coil. A dedicated small animal electrocardiographic triggering system (SA Instruments, USA) was utilized. Cine short axis images were acquired using a turbo field echo sequence (TFE) with the following parameters: repetition time 14 ms, echo time 4.5 ms, flip angle 30°, in-plane resolution 0.31 × 0.31 mm, image matrix 256 × 256, and slice thickness 2 mm, typically with 5 contiguous slices. The rats were anaesthesized with intramuscular administration of midazolam 0.15 mg/kg (Dormicum^®^, Roche; Grenzach-Wyhlen), Medetomidin 2 mg/kg (Dormitor^®^, Pfizer, Karlsruhe) and Fentanyl 0.005 mg/kg (Ratiopharm, Ulm). The heart rate was between 180 and 210 beats per minute during the scan. Endo- and epicardial contours were manually traced in end-diastole and endsystole in contiguous slices covering the whole left ventricle (LV) followed by calculation of ejection fraction using MunichHeart/MR [26,27]. For analysis of regional wall motion the left ventricular circumference was divided into 36 of equally sized sectors and percentage of wall thickening was calculated in each as the ratio of the difference of end-systolic and end-diastolic wall thickness to end-systolic wall thickness. For comparison with histology the left ventricular short axis slice showing the largest circumferential extent of myocardial infarct scar was aligned with the corresponding MRI slice (the slice showing lowest wall motion) using the intersections of the right ventricular walls and the inter-ventricular septum as landmarks. Infarct size was determined in histological sections as % of the LV endocardial circumference as described[28]. Based on histology, three areas of interest were defined: border zone including four sectors at both ends of the MI scar, infarcted area including the sectors involved by MI scar and the remote myocardium covering the rest of left ventricle. All analyses were done blinded by two independent investigators.

### Immunohistochemistry

Myocardial sections were stained with Masson's Trichrome staining (MT) for estimation of infarct size. Immunohistochemical staining was performed incubating sequential slides with mouse monoclonal antibodies against rat anti-alpha smooth muscle (clone 1A4, DakoCytomation, Carpinteria, USA) and anti-rat CD68 (Monoclonal mouse Anti-Rat CD68, Serotec, Dusseldorf, Germany) and staining was achieved using Fast Red Substrate system (DAKO #K0699, Hamburg, Germany) and Mayer's hematoxylin. Infarct size and localization was determined planimetrically in MT stained slides using Sigma Scan Pro 5 Image Analysis (Version 5.0, Aspire Software International, Ashburn, USA). Vessel density was analyzed after 4 weeks after actin staining using Axiovision 3.1 (Carl Zeiss Vision GmbH, Jena, Germany). In 8 randomly selected 20-fold magnified fields of the infarct border zone the percentage of vessels as fraction of complete field of view area was determined using S. CORE online image analysis (S.Co GmbH, Garching, Germany). Early monocytic CD68+ cell infiltration was analyzed after anti-rat CD68 staining after 3 days. The area of CD68+ cells as percentage of whole section area was determined planimetrically using Sigma Scan Pro 5 Image Analysis. Staining for human CD 31 cells and apoptotic cells was performed after staining with mouse monoclonal antibody against human CD 31 (Mouse Anti-Human CD31 monoclonal antibody, AbD Serotec, Dusseldorf, Germany) and Cy3 anti-mouse IgG (Invitrogen, Karlsruhe, Germany). Apoptotic cells were visualized with direct Fluorescein TUNEL labeling (Roche Diagnostics - Applied Science, Mannheim Germany). Nuclear DNA counterstaining was performed using VECTASHIELD, Mounting Medium with DAPI (Vector, Laboratories, Burlingame, USA). Double positive cells for human CD31 and TUNEL were counted in each section using fluorescence microscopy. Tissue sections were scored blindly by two independent investigators (S.K. and E.W.).

### Statistical analysis

Values in tables and figures are shown as means ± SEM. Differences were analyzed by the unpaired Student's t test or ANOVA as appropriate. P < 0.05 in the two-tailed test was considered statistically significant.

## Results

### Effects of EPO on MNC and eEPC apoptosis in vitro

**Figure 1:**
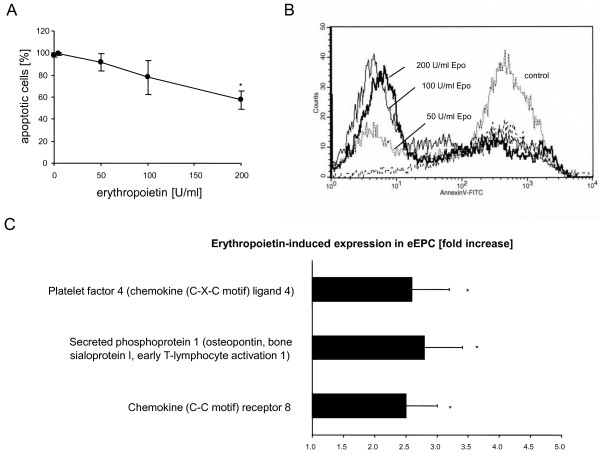
**Pretreatment with EPO dose dependently enhanced survival of leukocytes after serum starvation**. (a, b) Stimulation of eEPC with EPO induced enhanced expression of proangiogenic and proinflammatory factors. (c)

EPO dose-dependently reduced apoptosis in serum-starved leukocytes (Fig. 1A, B). This inhibition of apoptosis was associated with a decreased mRNA expression of the pro-apoptotic Bax in the presence of EPO (control: 0.65 + 0.09, EPO: 0.18 + 0.01, n = 4, P = 0.02). Similary EPO (200 U/l) decreased apoptosis in serum starved eEPC by 10 + 4% (n = 4) and decreased mRNA expression of Bax by 15%.

### Effects of EPO on inflammatory mediator expression in eEPCs

After stimulation with EPO, mRNA expression of Platelet factor 4 (chemokine (C-X-C motif) ligand 4), a strong chemoattractant for neutrophils and monocytes was increased 2.6 fold (p = 0.01) and chemokine receptor 8, involved in lymphocyte recruitment increased 2.7 fold, p = 0.04). Similarly stimulation of eEPC with EPO resulted in a 2.8 fold increase of secreted phosphoprotein 1, a cytokine involved in angiogenesis (Fig. 1C).

### Ejection fraction and regional wall thickening after permanent occlusion or temporary ligation by MRI

**Figure 2:**
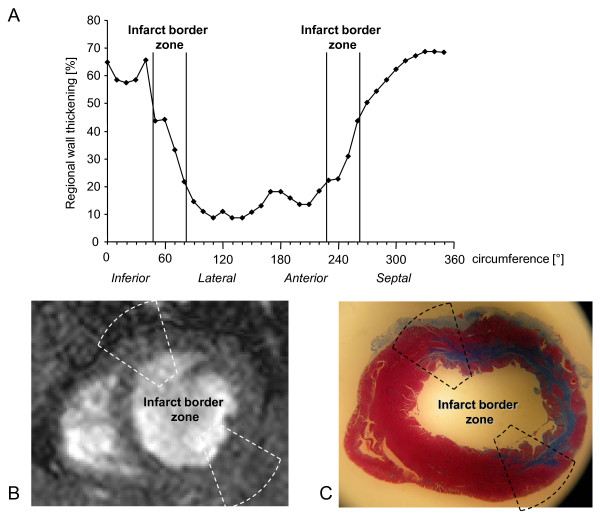
**Regional wall thickening measured in MRI of left ventricle and of infarct border zone (a) after alignment of MRI short axis plane (b) and histologic section (c)**.

To establish the method of regional wall thickening by MRI we compared the experimental myocardial infarction after temporary and permanent occlusion. Transient coronary occlusion resulted in medium size infarcts while permanent occlusion caused large infarcts as assessed by immunhistochemistry (I/R: 20 ± 4%, Lig 43 ± 4%, p = 0.001). In accordance ejection fraction by MRI was lower after permanent occlusion than after transient occlusion (Lig. 36 ± 4%, I/R: 48 ± 1%, p = 0.007). Regional wall thickening, calculated as ratio of the difference of end-systolic and end-diastolic wall thickness to end-systolic wall thickness, was determined in the anterolateral segments (Fig. 2). Values in sham operated rats were 92 ± 7%. Average wall thickening was reduced in rats with either transient or permanent ligation in the infarcted area: I/R 11 ± 7%, Lig. 3 ± 3%, the border zone of the infarction: I/R 14 ± 1%, Lig. 9 ± 2% and the remote myocardium I/R: 48 ± 17%, Lig. 17 ± 16% compared to sham operated rats (p < 0.001). After permanent or transient ligation wall thickening was less than 2 standard deviations of that seen in the sham operated animals in 98% and 92% of the infarct segments, in 90% and 92% of the border zone and 93% and 50% of the remote segments. Regional wall motion as percentage of systolic wall thickening was determined in the infarct border zone by matching histological sections stained with MT and short axis planes of MR imaging (Fig. 2). Regional function of the border zone of the infarction was significantly reduced in the permanent ligation group by 9 ± 2% compared to rats with transient ligation (14 ± 1%, p = 0.048). In sham operated animals we did not see changes in regional wall movement as compared to control animals. Thus regional wall thickening measured in the infarct border zone that reflects regional wall motion was improved in the transient ligation model.

### Effects of eEPCs and eEPCs with EPO on the ejection fraction and regional wall thickening after temporary ligation by MRI

**Figure 3:**
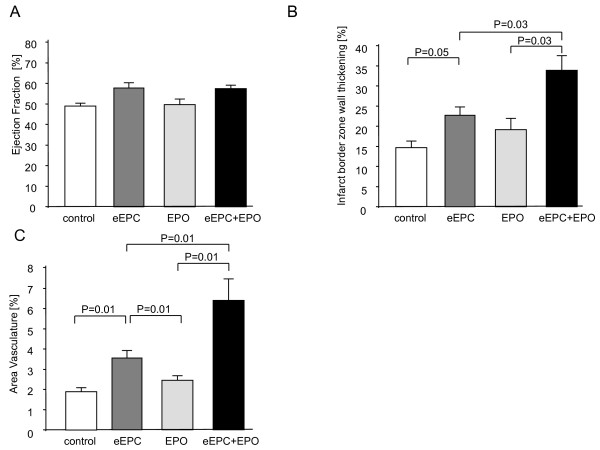
**No difference in global ejection fraction was seen between control and treatment groups**. (a) Application of eEPC + EPO results in an improved regional cardiac function in the infarct border zone (b) and results in increased neovascularization (c)

Regional wall thickening in the infarct border zone was significantly higher in rats that received eEPCs + EPO compared to eEPC, EPO alone or controls (Fig. 3B). Yet this improvement in regional wall motion did not translate into improvement of global systolic left ventricular function after four weeks (Fig. 3A). Moreover endsystolic and enddiastolic volumes were comparable in all groups (data not shown). Thus eEPCs in the presence of EPO as compared to eEPCs or EPO alone induce slight benefical effects on myocardial function that are reflected in improvement of regional wall movement.

### Immunohistology

Infarct size determined in MT-stained histological sections showed no significant differences after injection of eEPC: 18 ± 2%, EPO: 21 ± 3% or eEPC + EPO: 22 ± 2 (p = 0.77). Vascularization was determined after four weeks in sections stained for SMC actin. The number of vessels was significantly higher in the eEPC + EPO group as compared to eEPC (6.5 ± 1% vs. 3.6 ± 0.4%; p = 0.01). In comparison with the EPO and the control group the eEPC group showed a significantly increased vascularization in the infarct (Fig 3C).

**Figure 4:**
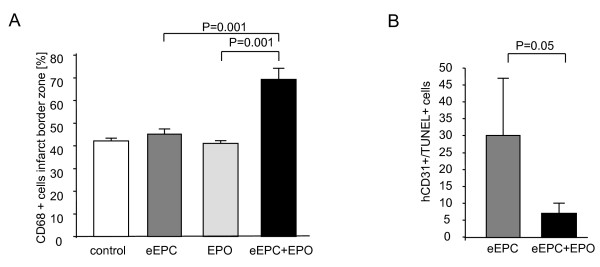
**Application of eEPC + EPO enhances recruitment of CD68+ cells to the infarct border zone (a) and reduces the number of apoptotic eEPCs (b) determined as double positive for human CD31 and TUNEL in immunofluorescence**.

Recruitment of inflammatory cells to the border zone of the infarction was evaluated 3 days after myocardial infarction by staining for mononuclear CD68+ cells. Quantitative analysis revealed a significantly higher proportion of CD68+ cells in the eEPC + EPO group compared to eEPC, EPO alone or controls, indicating an increased inflammation (Fig 4A)

After 3 days the number of transplanted cells showing signs of apoptosis, measured as double positive for TUNEL and human CD31 staining was significantly reduced in rats that were treated with eEPCs + EPO as compared to treatment with eEPCs (7 ± 3% vs. 30 ± 3%; p = 0.05). (Fig. 4B)

## Discussion

The main findings of this study are: (1.) EPO inhibited apoptosis in leukocytes and eEPCs. (2.) Regional wall thickening was improved after treatment with eEPCs + EPO as compared to eEPCs, EPO alone or PBS (control). This was associated with an increase in inflammatory infiltrate and surviving eEPCs 3 days after myocardial infarction and with an increase in vasculogenesis after 4 weeks.

In a recent study we have shown that eEPC improve global ejection fraction after intramyocardial transplantation [1]. To evaluate if additional EPO may further improve myocardial function assessed by MRI these experiments were performed. Previous studies showed anti apoptotic effects of EPO on various cell types, such as endothelial cells and cardiomyocytes[10-12,14]. We confirmed these anti-apoptotic effects in leukocytes and eEPCs in vitro. Besides these anti-apoptotic effects, additional biological functions of EPO on inflammatory or immune pathways may occur. Previous studies showed that EPO enhances cellular and humoral components of the immune system and EPO stimulation of dendritic cells leads to improved functionality and maturation[29-32]. We demonstrated an enhanced expression of lymphocyte and monocyte attractants in eEPCs after stimulation with EPO. This proinflammatory effect was combined with an enhanced expression of proangiogenic Platelet factor 4. Taken together EPO stimulation of eEPC altered the inflammatory expression profile, which could potentially alter myocardial remodelling by recruitment of inflammatory cells and enhanced angiogenesis.

Previous studies mainly examined systemic administration of EPO in experimental myocardial infarction and observed improved survival, cardiac function and enhanced progenitor cell mobilization and homing[10-12,24]. Intramyocardial application of EPO in a model of permanent ligation of the left anterior descending artery in rats revealed an improved left ventricular function in pressure volume loops, a histological smaller infarct size and greater capillary density[33-35]. These effects could not been confirmed in the setting of ischemia and reperfusion in our study, which might be due to smaller infarctions due to early reperfusion and different dosages of intra-myocardial injected EPO.

Local application of eEPCs have been shown to improve myocardial function by enhancing vasculogenesis [1]. In the presence of EPO transplantation of eEPCs improved regional function even further compared to eEPCs alone. Injection of eEPC and EPO was associated with increased CD68+ inflammatory cell recruitment and enhanced vessel formation. As a possible mechanism the effect of EPO on the transplanted eEPC, with increased expression of molecules that are involved in angiogenesis and recruitment of inflammatory cells could be hypothesized. In addition local EPO may inhibit leukocyte apoptosis. Finally, EPO improves the survival of the transplanted eEPCs within the border zone of the myocardial infarction.

Cardiac function was analyzed using MR imaging. This approach was established using a permanent and temporary ligation model. Regional wall function measured in MRI proofed to be a valid and a more sensitive parameter than global ejection fraction. Alignment of MRI with histologic infarct areas was reproducible and could reveal differences that where not detected by measurement of global ejection fraction. Histologic sections were used for alignment instead of late enhancement MRI measurements, because of higher accuracy in the identification of often small and non-transmural infarctions.

The proposed mechanisms of cardiac improvement by cell transplantation in experimental myocardial infarction are paracrine effects and enhanced vascularization. Improvement of regional wall function in the infarct border zone was observed in rats that received eEPC + EPO without changing the global ejection fraction or infarct size. This regional effect was associated with an increase of paracrine activity of the transplanted cell presumably due to EPO stimulation and seemed not sufficient to further increase global systolic function or infarct size on top of cell transplantation. Previous studies suggest that regenerative effects of cell therapy are the more pronounced the bigger the infarction is. The mainly non-transmural infarctions in this animal model might be too small to detect any effect concerning ejection fraction or infarct size and point to the relevance of more sensitive regional measurements.

In summary transplantation of eEPCs + EPO improves regional wall thickness by MRI and is associated with improved eEPC survival, increased inflammatoriy cell infiltrate and vascularisation. Anti-apoptotic and immune modulatory effects of EPO may contribute to this effect. Thus EPO in addition to cell therapy may prove benefical to improve myocardial remodeling.

## Abbreviations

eEPC: expanded endothelial progenitor cells; EPO: erythropoietin; MI: Myocardial infarction; MRI: Magnetic Resonance Imaging.

## Competing interests

The authors declare that they have no competing interests.

## Authors' contributions

AS carried out the MR studies, the imunhistochemistry, the in vitro experiments, he performed the statistical analysis and wrote the manuscript. MK, SK, MS and EW participated in the animal experiments and the immunohistochemistry. MM, SK, AK, PG, AS and RB participated in the MR imaging and analysis. RAJ participated in the generation of eEPCs. IO conceived of the study, participated in its design and coordination, performed the statistical analysis and wrote the manuscript. All authors read and approved the manuscript.

## Pre-publication history

The pre-publication history for this paper can be accessed here:

http://www.biomedcentral.com/1471-2261/10/43/prepub
